# Inducible Protective Processes in Animal Systems XIII: Comparative Analysis of Induction of Adaptive Response by EMS and MMS in Ehrlich Ascites Carcinoma Cells

**DOI:** 10.1155/2014/703136

**Published:** 2014-06-04

**Authors:** Periyapatna Vishwaprakash Mahadimane, Venkateshaiah Vasudev

**Affiliations:** Department of Studies in Bioscience, Post-Graduate Centre, University of Mysore, Hemagangotri, Hassan, Karnataka 573220, India

## Abstract

In order to investigate the presence of adaptive response in cancerous cells, two monofunctional alkylating agents, namely, ethyl methanesulfonate (EMS) and methyl methanesulfonate (MMS), were employed to treat Ehrlich ascites carcinoma (EAC) cells *in vivo*. Conditioning dose of 80 mg/kg body weight of EMS or 50 mg/kg body weight of MMS and challenging dose of 240 mg/kg body weight of EMS or 150 mg/kg body weight of MMS were selected by pilot toxicity studies. Conditioned EAC cells when challenged after 8 h time lag resulted in significant reduction in chromosomal aberrations compared to challenging dose of respective agents. As has been proved in earlier studies with normal organisms, even in cancerous cells (EAC), there is presence of adaptive response to methylating and ethylating agents. Furthermore, it is also interesting to note in the present studies that the methylating agent, MMS, is a stronger inducer of the adaptive response than the ethylating agent, EMS.

## 1. Introduction

An important and essential characteristic of organisms is their ability to survive in face of repeated exposure to different physical and chemical genotoxic agents. Adaptive response is one of many defense mechanisms that have evolved to minimize genotoxic damages. In 1977, Samson and Cairns [1] demonstrated this phenomenon in* E. coli* and they showed that a response was induced by a low level of MNNG that enabled cells both to survive and to be less mutated by subsequent high toxic dose of MNNG than control cultures. After this discovery, extensive work has been done by using prokaryotes and* in vitro* and* in vivo* eukaryotes [2–11] including* in vitro* human lymphocytes [12–20]. Reports are available on the existence of such phenomenon even in higher plants [21–23]. Our laboratory also reported cytogenetic adaptive response in* in vivo* animal systems such as grasshopper* Poecilocerus pictus* [24–27] and mouse [28–32].

From several recent studies it is also interesting to note the presence of adaptive response not only in normal cells but also in cancer cells. Scrutiny of reports has demonstrated the protection against radiations in human malignant melanoma, epidermoid laryngeal carcinoma, ovarian carcinoma, myeloma, fibrosarcoma, glioblastoma, and epithelial carcinoma [33–37], but only a very few reports are available in chemically induced adaptive response [38–40]. Contrary to these results, Park et al. [41] using mouse papilloma and lymphoma, Schaffer et al. [42] using human bladder carcinoma, Jiang et al. [43] using two leukemia cell lines (erythroleukemia, promyelocytic leukemia) and two solid tumor cell lines (lung carcinoma and glioma), Sowa et al. [44] using colon carcinoma, and Wang et al. [45] using gastric cancer cells have clearly showed the absence of the adaptive response. Thus, there are conflicting reports on the adaptive response in cancerous cell. Further, as far as the authors are aware, in almost all the studies in cancer cells, the adaptive response has been assessed on the basis of cell survival. No attempt has been made by using cytogenetic assay to determine the adaptive response on one hand and, on the other hand, there are also no sufficient reports on adaptive response induced by chemical agents in cancerous cells. This has prompted us to study the influence of alkylating agents on adaptive response in cancerous cells and results are presented here.

## 2. Materials and Methods

### 2.1. Chemicals

Alkylating agents, methyl methanesulfonate (MMS) (CAS number 66-27-3), and ethyl methanesulfonate (EMS) (CAS number 62-50-0) were obtained from Sigma Co., St. Louis, MO, USA, and colchicine (CAS number 64-86-8) was obtained from Himedia, Pvt. Ltd., Mumbai, India. Giemsa stain and other chemicals of analytical grade were commercially available. EMS and MMS were dissolved in 0.9% NaCl to obtain the required concentrations. Freshly prepared solutions of these agents were used each time. The conditioning and challenging doses of EMS (80 and 240 mg/kg body weight) and MMS (50 and 150 mg/kg body weight) were selected from the earlier experiments [28, 29]. Even though these were selected from treated normal animals, the effect of these along with the range (namely, 25–160 mg/kg body weight of MMS and 50–300 mg/kg body weight of EMS) of doses was analyzed in EAC cells by preliminary pilot toxicity studies.

### 2.2. Animals

Male Swiss albino mice weighing 25–30 g of 6–8 weeks old were used and housed in polypropylene cages which were provided with standard feed pellets and water* ad libitum* under 12 h of light/dark cycle. Study was approved by the Institutional Animal Ethical Committee according to the institutional guidelines and the national animal welfare regulations.

### 2.3. Tumor Cells

Animals with the Ehrlich ascites carcinoma (EAC) cells were initially obtained from Department of Applied Zoology, Mangalore University, Mangalore, India, and EAC cells were maintained by weekly intraperitoneal (*i.p.*) inoculation of 10^6^ cells/mouse.

### 2.4. Treatment Schedule

Each animal was inoculated with 0.2 mL of saline containing 1 × 10^6^ EAC cells and this day was taken as zero day. On the tenth day after inoculation, 0.5 mL of saline containing conditioning or challenging doses of EMS or MMS or 0.5 mL of saline only was injected* i.p.* into animals. For combined treatment, 8 h time lag (TL) between conditioning and challenging doses was selected from the previous experiments by the authors [28, 29], who have shown that an 8 h time lag has offered the maximum protection with respect to chromosomal aberrations in mouse bone marrow cells compared to other time lags. 24 h, 48 h, and 72 h recovery times have been employed for all groups for chromosome analysis.

### 2.5. Chromosome Analysis

Each animal received 0.5 mL of 0.05% colchicine by* i.p.* injection 90 minutes prior to the removal of ascites. 0.1 mL of ascitic fluid was removed at 24 h, 48 h, and 72 h recovery times. This was processed and slides were prepared by modified method of Evans et al. [46]. In brief, 0.1 mL ascitic fluid was added to 0.4 mL of 0.3% NaCl and this was incubated at 37°C for 45 minutes. After incubation, the mixture was centrifuged at 1000 rpm for 10 minutes and the supernatant was discarded. To the pellet, 5 mL of fixative (3 : 1 v/v of ethanol : acetic acid) was added and mixed intensively to avoid clumping of cells. The tubes were kept at 4°C for 30 minutes and then centrifuged at 2000 rpm for 5 minutes. The pellet was processed thrice as above. Finally the pellet was resuspended in 0.5 mL of fixative, dropped on to clean, nongreasy, prechilled slides, and heat fixed. Coded Giemsa stained slides were screened for presence of chromosomal aberrations such as chromatid breaks, exchanges, triradials, intrachromatid deletion, isochromatid deletion, dicentrics, rings, and minutes and tabulated. In each treatment group, a minimum of 300 well-spread, nonoverlapping metaphase plates was scored and a minimum of three experiments was conducted for all recovery times.

### 2.6. Statistical Analysis

The data obtained from the experiments were subjected to statistical analysis to determine the significance level between the control and treatment groups. The difference that exists among the treatment groups was analyzed using the Duncan multiple comparison post hoc test using the SPSS software (version 17.0). Breaks/cell was calculated as per the method of Savage [47].

## 3. Results and Discussion

Alkylating agents are one of the extensively used drugs for cancer chemotherapy for long-term treatment [48–50]. Among these agents, monofunctional methylating and ethylating agents are prominent in these days of cancer protection [51, 52] because of their clastogenic and mutagenic potency in varied genetic systems [53–58]. Clastogenic effects of both EMS and MMS have been well documented even in cancerous cells [59–61]. For the first time, using chromosomal analysis, Mahadimane and Vasudev [62] showed induction of aberrations in EAC cells by MMS and these studies with that of Lettré et al. [63] have suggested exploiting this cell line for clastogenic adaptive response.

**Figure 1:**
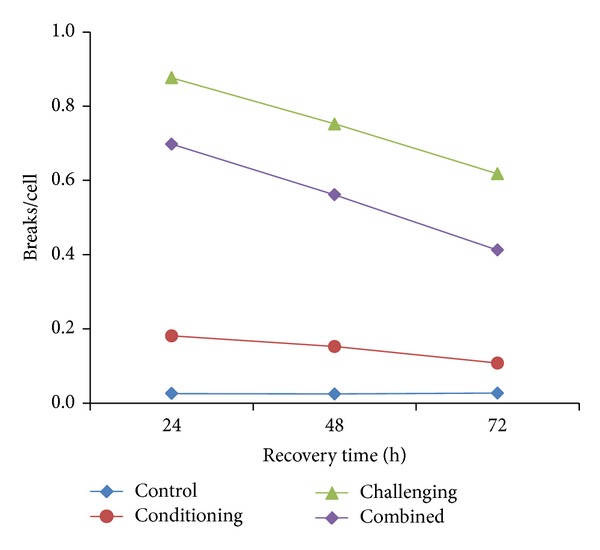
Yield of breaks/cell after different treatment schedules of EMS.

**Figure 2:**
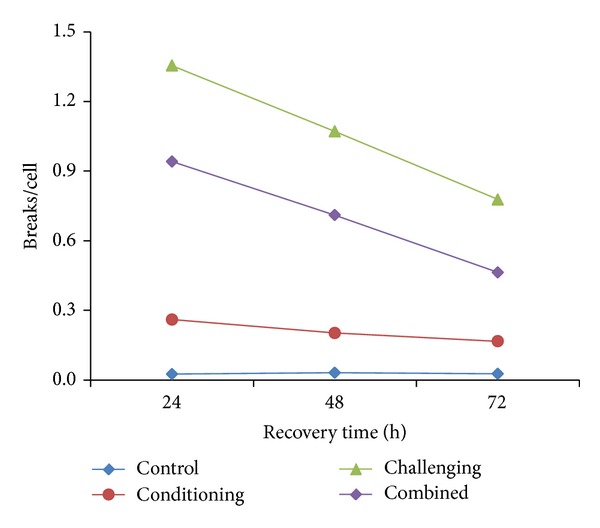
Yield of breaks/cell after different treatment schedules of MMS.

S-dependent nature of EMS and MMS by inducing mostly chromatid type of aberrations in EAC cells (Tables 1, 2, and 3) corresponds to similar effects of these agents in different test systems [64, 65]. This is also true with reference to the induction of chromosomal aberrations in EAC cells irrespective of different recovery times (Tables 1–3). It is also clear from the these tables that even though low dose of EMS (80 mg/kg body weight) produces a significant level of aberrations compared to controls, a dose as less as 50 mg/kg body weight of MMS is sufficient to induce still more aberrations compared to EMS. This is also true with challenging dose in EAC cells where the yield of breaks/cell after exposing to EMS and MMS was 0.91 ± 0.04 and 1.35 ± 0.06, respectively, at 24 h RT (Figures 1 and 2). These values are highly significant compared to control (*P* < 0.01). Similarly Rao and Natarajan [64] using* Vicia faba*, Vogel and Natarajan [65] using* Drosophila*, Riaz Mahmood et al. [29] using mouse, and Harish et al. [19] using human lymphocytes have shown that MMS is more potent inducer of aberrations than EMS and concluded that methylating agents are strong inducers of chromosomal aberrations compared to ethylating agents in different test systems.

In order to understand the peak activity of adaptive response, we have earlier tested different time lags between conditioning and challenging doses in mouse and concluded that the reduction in the number of aberrations is a consequence of high activity of repair enzymes in the cells that were more adaptive for the 8 h time lag than others [29]. The same time lag was employed in the present investigations. Pretreatment of low dose of EMS or MMS and then treated with high dose of the same agent after 8 h time lag resulted in significantly reduced frequency of chromosomal aberrations in EAC cell (*P* < 0.05). These results confirm the earlier observations of the authors [29]. This is the first report on the induction of adaptive response using chemical agents in* in vivo* cancer system using clastogenic end point, as far as our knowledge goes.

The preliminary toxicity studies in EAC cells with MMS have revealed that there is dose effect relationship. Even though the tested lowest dose of 25 mg/kg body weight could not induce significant chromosomal aberration compared to controls, the doses of 50 mg/kg body weight and above produced significant aberrations [61]. Similarly, in case of EMS, doses above 80 mg/kg body weight yielded significant aberrations (unpublished data).

The present* in vivo* results of EAC cells are consistent with those of Lee et al. [40], where they have demonstrated the presence of adaptive response in* in vitro* sarcoma 180 cells primed with low dose of EMS using survival of the cells as the end point. Similarly employing radiations (low dose of 0.05 cGy/day for 4-day period) Boothman et al. [33] showed adaptive response in human malignant melanoma cells.

Shadley et al. [66] suggested that the response ceases after the third mitosis of adapted cells, due to dilution of the repair system as the cells divide into subsequent cell cycles. Similarly, it was earlier shown [29–32] that, at 72 h RT, third subsequent mitoses, the reduction in frequency of chromatid aberration is very minimal in normal mouse bone marrow cells. Another point they also noted in the same system is that the lowest aberration frequency was observed at 72 h RT compared to 24 h and 48 h RTs. In the present investigation, it is observed that, at 72 h RT in EAC cells, that is, cancerous cell, there is reduction in chromatid aberrations and it can also be noted that the lowest aberrations were seen at 72 h than at 24 and 48 h RT (Tables 1–3). This agrees with earlier reports where it has been amply proven that the decrease in aberration frequency with increasing culturing time reflects a mechanism of mitotic selection of aberration bearing cells [67]. When the data of normal and cancer cells are taken together with respect to the adaptive response and different recovery times, there is not much difference.

The decline in aberration frequency was evident in that it produced 57.89% when the animals were treated with both conditioning and challenging doses separated by 8 h (combined treatment) compared to 101.44% of additive effect of both the conditioning and challenging doses of MMS treated at 24 h RT (Table 4). This reduction in induced aberration frequencies by the combined treatment of MMS or EMS was observed over the whole range of RTs tested (Table 4). These results suggest that the conditioning doses of MMS or EMS given 8 h before the challenging treatment might have induced protection against the damaging effects of the challenging dose of respective agents in the* in vivo* EAC cells. It is pertinent to mention here that EMS or MMS induces adaptive response by significantly repairing the damaged meiotic cells of* P. pictus* and bone marrow cells of mouse [24–32]. Thus it appears from the results that both categories of alkylating agents, ethylating EMS and methylating MMS, possess the ability to induce an adaptive response in* in vivo* system of EAC cells similar to normal cells. However, low dose of MMS is more effective in conditioning the mitotic cells of EAC than EMS. Thus, maximum reduction from 56.89% to 26.22% (about 53.91%) can be seen in combined treated EAC cells by MMS at 72 h RT, whereas, in the case of EMS, the maximum reduction of aberrations is from 52.44% to 30.44% (about 41.94%) at the same recovery time (Table 4). It appears that both chemicals induce adaptive response, but the methylating agent is a more effective inducer. Similar results were also observed in the induction of adaptive response in* in vivo* mouse test system at 8 h time lag [29–32]. These results are supported by the finding of Olsson and Lindahl [68], where they have demonstrated that the* Ada* coded methyltransferase transfers the ethyl group O^6^-ethylguanine at a rate of 10 times less than that for the methyl transfer. These are further strengthened from the evidences presented by earlier researchers [69, 70]. In* in vivo* wild-type* E. coli*, the adaptive response began to contribute to O^6^-methylguanine repair about one hour after alkylation, which is the time required for full induction of the* Ada* DNA methyltransferase. In contrast, the adaptive response did not play such a large role in the repair of O^6^-ethylguanine and O^4^-ethylthymine, presumably because DNA ethylation damage is a poor inducer of the adaptive response. These observations including the present* in vivo* EAC cells studies underline the importance of ethylation and methylation in the analysis of adaptive response and hence further work is required in this direction.

In summary, based on the present results and our previous studies it can be concluded that both EMS and MMS induce adaptive response not only in normal cells but also in cancerous cells. Further, there is also indication of the existence of differential adaptive response to chromosome damage induced by ethylating and methylating agents in* in vivo* EAC cells, similar to that described in normal biological systems. Taking into account that chemotherapy with alkylating drugs is based on their genotoxic and clastogenic efficiency to interrupt replication and tumor cell division, what prospects are for clinical implications of the adaptive response in such cells? Probably, differences between clastogenic effects of methylating and ethylating agents and their capacity to trigger the protective mechanism against chromosomal aberrations in tumor cells might be important for elaboration of chemotherapy approaches. But, in the context of clinical applications, the fact is more interesting that inducible O^6^-methylguanine-DNA methyltransferase has been recognized as a potential target for cross-linking agents causing enzymatic depletion to increase susceptibility of tumor cells to action of other alkylating drugs [71, 72].

## Figures and Tables

**Table 1:** Frequency of chromosomal aberrations observed after conditioning, challenging, and combined treatments of EMS in EAC cells at 24 h recovery time.

Treatment	Chromosomal aberrations									Total aberrations
	Exp. number	B′	B′′	RB′	RB′B′′	Minutes	Dic	Rings	ID	
Control	1	8	—	—	—	1	—	—	—	9 (3.00)
	2	7	—	—	—	1	—	—	—	8 (2.67)
	3	6	—	—	—	—	—	—	—	6 (2.00)
	Mean ± SE	7.00 ± 0.58	—	—	—	0.67 ± 0.33	—	—	—	7.67 ± 0.88^a^ (2.56)

Conditioning	1	22	4	4	3	8	—	—	4	45 (15.00)
	2	23	3	3	3	6	1	—	2	41 (13.67)
	3	24	3	2	2	5	—	—	5	41 (13.67)
	Mean ± SE	23.00 ± 0.58	3.33 ± 0.33	3.00 ± 0.58	2.67 ± 0.33	6.33 ± 0.88	0.33 ± 0.33	—	3.67 ± 0.88	42.33 ± 1.33^b^ (14.11)

Challenging	1	95	15	20	23	29	3	2	12	199 (66.33)
	2	86	13	21	21	20	2	1	8	172 (57.33)
	3	82	17	22	18	24	2	1	9	175 (58.33)
	Mean ± SE	87.67 ± 3.84	15.00 ± 1.15	21.00 ± 0.58	20.67 ± 1.45	24.33 ± 2.60	2.33 ± 0.33	1.33 ± 0.33	9.67 ± 1.20	182.00 ± 8.54^c^ (60.67)

Combined	1	65	11	19	20	20	2	1	8	146 (48.67)
	2	72	10	17	18	19	1	1	6	144 (48.00)
	3	68	10	20	15	16	2	1	5	137 (45.67)
	Mean ± SE	68.33 ± 2.03	10.33 ± 0.33	18.67 ± 0.88	17.67 ± 1.45	18.33 ± 1.20	1.67 ± 0.33	1.00 ± 0.00	6.33 ± 0.88	142.33 ± 2.73^d^ (47.44)

Note: data of 3 independent experiments; 3 animals per experiment were used; 100 cells per animal scored; and a total of 900 cells scored per dose. B′: chromatid break, B′′: isochromatid break, RB′′: chromatid exchange, RB′B′′: triradials, Dic: dicentrics, and ID: intrachromatid deletion. Values with same superscripts are not significantly different (*P* > 0.05), whereas values with different superscripts are significantly different (*P* < 0.05) from one another. Parentheses show the percentage of total aberrations.

**Table 2:** Frequency of chromosomal aberrations observed after conditioning, challenging, and combined treatments of EMS in EAC cells at 48 h and 72 h recovery times.

Recovery time (h)	Treatment	Chromosomal aberrations		Total aberrations
		Chromatid type	Chromosome type	
48	Control	7.33 ± 0.67	—	7.33 ± 0.67^a^ (2.44)
	Conditioning	32.67 ± 0.88	3.00 ± 0.58	35.67 ± 1.45^b^ (11.89)
	Challenging	143.00 ± 5.51	14.33 ± 2.03	157.33 ± 7.31^c^ (52.44)
	Combined	106.67 ± 1.86	11.67 ± 1.76	118.33 ± 2.96^d^ (39.44)

72	Control	8.00 ± 1.16	—	8.00 ± 1.16^a^ (2.67)
	Conditioning	25.33 ± 2.33	1.33 ± 0.33	26.66 ± 2.60^b^ (8.89)
	Challenging	118.67 ± 1.86	12.00 ± 1.53	130.67 ± 2.96^e^ (43.56)
	Combined	84.00 ± 3.79	7.33 ± 1.20	91.33 ± 4.98^f^ (30.44)

Note: average data of 3 independent experiments; 3 animals per experiment were used; 100 cells per animal scored; and a total of 900 cells scored per dose. Values with same superscripts are not significantly different (*P* > 0.05), whereas values with different superscripts are significantly different (*P* < 0.05) from one another. Parentheses show the percentage of total aberrations.

**Table 3:** Frequency of chromosomal aberrations observed after conditioning, challenging, and combined treatments of MMS in EAC cells at 24 h, 48 h, and 72 h recovery times.

Recovery time (h)	Treatment	Chromosomal aberrations		Total aberrations
		Chromatid type	Chromosome type	
24	Control	7.67 ± 0.67	—	7.67 ± 0.67^a^ (2.56)
	Conditioning	49.00 ± 4.58	5.00 ± 1.53	54.00 ± 4.62^c^ (18.00)
	Challenging	217.33 ± 5.36	33.00 ± 3.06	250.33 ± 7.88^h^ (83.44)
	Combined	152.67 ± 4.33	21.00 ± 2.08	173.67 ± 6.39^f^ (57.89)

48	Control	9.00 ± 1.00	—	9.00 ± 1.00^a^ (3.00)
	Conditioning	39.67 ± 2.60	3.00 ± 0.58	42.67 ± 2.40^bc^ (14.22)
	Challenging	172.00 ± 2.00	23.00 ± 1.15	195 ± 3.06^g^ (65.00)
	Combined	119.00 ± 6.43	13.00 ± 1.53	132 ± 7.09^e^ (44.00)

72	Control	8.00 ± 1.53	—	8.00 ± 1.53^a^ (2.67)
	Conditioning	34.67 ± 0.88	2.67 ± 0.33	37.33 ± 0.67^b^ (12.44)
	Challenging	120.67 ± 4.41	12.67 ± 1.86	133.33 ± 2.91^e^ (44.44)
	Combined	69.00 ± 2.89	9.67 ± 1.46	78.67 ± 3.93^d^ (26.22)

For explanations, see Table 2.

**Table 4:** Percent reduction of chromosomal aberrations in EAC cells after combined treatment of EMS or MMS at different recovery times.

Alkylating agent	Recovery time (h)	*A* Additive effect (conditioning + challenging dose) (%)	*B* Combined effect (treatment of conditioning and challenging dose together) (%)	*C* Reduction (%)
MMS	24	101.44	57.89	42.93^b^
	48	79.22	44.00	44.46^b^
	72	56.88	26.22	53.91^c^

EMS	24	74.78	47.44	36.55^a^
	48	64.33	39.44	38.69^b^
	72	52.45	30.44	41.95^b^

Note: values with the same superscripts are not significantly different (*P* > 0.05), whereas values with different superscripts are significantly different (*P* < 0.05) from one another. Percentage of reduction was calculated by using formula *C* = (*B*/*A*∗100) − 100.
